# Effects of gamification on EFL learning: a quasi-experimental study of reading proficiency and language enjoyment among Chinese undergraduates

**DOI:** 10.3389/fpsyg.2025.1448916

**Published:** 2025-03-18

**Authors:** Jing Cheng, Chen Lu, Qiaoling Xiao

**Affiliations:** School of International Education, Wuhan University of Science and Technology, Wuhan, Hubei, China

**Keywords:** gamification, EFL learning, language enjoyment, reading proficiency, student engagement, classroom teaching

## Abstract

**Background:**

In foreign language education, educators struggle with declining student engagement as traditional EFL teaching, relying on passive lectures and dull materials, hampers proficiency and dampens passion. Gamification has emerged as a potential solution. This quasi-experimental study, based on the broaden-and-build theory, examined the effects of gamification on reading proficiency and foreign language learning enjoyment (FLLE) among Chinese undergraduates studying English as a foreign language (EFL).

**Methods:**

Data were collected from 220 first-year undergraduates at a Chinese university through reading assessments and the Chinese Foreign Language Enjoyment Scale, supplemented by interviews with nine participants picked from the first-year undergraduates.

**Results:**

The findings revealed a significant increase in gamification's benefits for EFL reading proficiency. FLLE's private dimension, tied to personal enjoyment, was crucial. Additionally, gamified settings improved focus, teamwork, and communication.

**Discussion:**

This study supports integrating gamification to boost engagement and outcomes. However, the study was limited to a specific context and duration. Therefore, future studies should identify key gamification elements and their long-term impact.

## 1 Introduction

Educators have long struggled with declining student engagement in the realm of foreign language education. Traditional lecture-style learning often fails to maintain student curiosity and attention (Wang, 2022b). This is especially pronounced in English as a foreign language (EFL) instruction. Educational methodologies have recently shifted toward interactive approaches. Gamification, which incorporates game design elements into traditional learning environments, has emerged as a promising solution. These strategies complement traditional EFL methodologies effectively (Cruz et al., 2023; Hamari et al., 2014; Kapp, 2012). Previous studies have demonstrated that gamification benefits EFL learners by improving their language skills, such as vocabulary, grammar, pronunciation, reading, and speaking, and boosting their intrinsic motivation and engagement. For instance, Zhang and Hasim (2023) found that gamification cultivates comprehensive literacy by providing an authentic learning environment and collaborative opportunities for applying language skills.

Foreign language learning enjoyment (FLLE), central to language acquisition, has gained prominence with positive psychology. Previous studies have demonstrated that enjoyable learning experiences can enhance students' motivation, self-efficacy, and wellbeing in foreign language learning (Dong et al., 2022). When students find pleasure in language tasks, they are more likely to actively engage in the learning process (Wang, 2022a). Moreover, FLLE underscores the importance of positive emotional engagement in learning, positively impacting students' foreign language achievements (Dewaele and Alfawzan, 2018; Piechurska-Kuciel, 2017). However, the dynamics of FLLE within gamified environments—and, by extension, foreign language proficiency—remain largely unexplored, with a dearth of research focusing explicitly on this connection, as exemplified by a preliminary yet insightful study by Liu et al. (2024).

Empirical evidence supports exploring gamification's impact on EFL learning. However, research on these effects on reading proficiency and FLLE remains limited. Since reading proficiency is a cornerstone of language acquisition that necessitates cognitive engagement as well as continuous motivation and positive emotional experiences (Al-Obaydi et al., 2024; Guthrie and Klauda, 2014), it is imperative to delve into the intricacies of FLLE in the context of reading within gamified EFL environments. Such an exploration is valuable, as it can potentially uncover how the synergy between enjoyment and achievement in reading can be effectively utilized to bolster overall language proficiency by incorporating gamified techniques. This understanding is crucial in enhancing English outcomes in Chinese education based on the authoritative *College English Teaching Guide* regulated by China's Ministry of Education (The National Administry Committee on Teaching English Language in Higher Education under the Ministry of Education, 2020).

This study examines gamification's impact on reading proficiency and FLLE among Chinese undergraduate EFL learners. Anchored in the broaden-and-build theory, it seeks to elucidate how gamification contributes to or detracts from learning experiences and outcomes. This research highlights gamification's role in improving language strategies, reading proficiency, and FLLE.

## 2 Literature review

This section discusses research on the broaden-and-build theory in foreign language learning, provides an overview of studies on FLLE, and introduces studies on gamification in foreign language learning. This study aims to understand how gamification impacts language learning.

### 2.1 Broaden-and-build theory

Introduced by Barbara Fredrickson in 1998 and expanded in 2001, the broaden-and-build theory suggests that positive emotions do more than evoke positive emotions and widen our array of thoughts and actions; consequently, this builds a vast array of enduring personal resources, ranging from physical and intellectual to social and psychological assets (Fredrickson, 1998; Cohn et al., 2009; Lyubomirsky et al., 2005). While central to psychology, this theory's relevance to language learning is newly recognized. Positive emotions improve cognitive processes in language acquisition (Gregerson et al., 2014; MacIntyre and Gregersen, 2012). These processes include improved attention and memory for language processing into learners' existing linguistic frameworks (Pekrun et al., 2011; Swain, 2013).

This study focuses on learners' enjoyment and engagement, which aid language skill acquisition (Lake, 2013). Further, Jin and Zhang (2019) stated that enjoyment helps learners build language resources and broaden their perspectives. Academic engagement reflects a learner's energy, enthusiasm, and focus (Hiver et al., 2021; Ronnel et al., 2015). Enjoyment and engagement are essential for foreign language success (Jin and Zhang, 2021); this illustrates how positive emotions expand thoughts and actions, enhancing language acquisition and retention (Fredrickson and Branigan, 2005).

Another critical dimension taps into the learning environment, as a more positive and engaging learning environment can broaden learners' perspectives and build cognitive resources, leading to more effective learning (Oxford, 2016). Leung et al. (2019) demonstrated that such environments reduce anxiety and promote communication. Therefore, teachers are encouraged to create a positive learning environment through respect and care for students, which increases learners' interest in foreign languages (Wang et al., 2021). However, research rarely examines how gamified classrooms foster positive emotions to establish learners' physical, psychological, intellectual, and social resources.

### 2.2 Foreign language learning enjoyment

With the broaden-and-build theory, FLLE represents learners' positive emotional encounters (Dewaele and MacIntyre, 2014). In foreign language classrooms, there are different kinds of challenges related to language skills, and when skills match the level of challenge, enjoyment may arise (Csikszentmihalyi, 1975). FLLE reflects the balance between challenge and self-competence, which mirrors a person's innate motivation to succeed when confronted with arduous tasks (Dewaele and MacIntyre, 2016). This definition illustrates that FLLE expands an individual's immediate range of thoughts and behaviors while fostering personal wellbeing and long-term development (Fredrickson, 2001). For language learners, enjoyment and playfulness benefit language learners significantly because play has been linked to promoting social connections and brain development (Dewaele and MacIntyre, 2014).

FLLE is a dynamic construct interacting with different variables within foreign language-learning processes. Extraverts report higher FLLE than introverts (Pan and Zhang, 2021). Teachers' positive traits, such as amiability, enthusiasm, and sense of humor, can also positively influence FLLE (Dewaele et al., 2019; Dewaele and Li, 2021; Dewaele et al., 2022). An enthusiastic teacher can create a more engaging atmosphere, enhancing the students' FLLE (Dewaele and Li, 2021). Moreover, Dewaele and MacIntyre (2019) found that FLLE was influenced by their degree of cultural empathy. This finding contributes to the literature by proving that FLLE is relevant across different cultural contexts.

Among learning outcomes, a wealth of empirical research has consistently revealed that FLLE closely relates to academic achievement, classroom participation, and the willingness to communicate (WTC). High FLLE correlates with better academic performance (Li et al., 2019). Botes et al. (2022) further confirmed this positive correlation in a meta-analysis. This association underscores enjoyment as a motivator in learners to invest more effort in language studies. Furthermore, increased academic engagement can be found with a higher level of FLLE (Wang, 2022a), and learners who enjoy the language-learning process are more likely to engage in communication in the target language (Li et al., 2022a).

Although foreign language educational literature has extensively examined FLLE's essential role, a majority have explored this as a unidimensional construct; only a few studies have investigated FLLE's factorial structure in foreign language-learning contexts. Dewaele and MacIntyre (2014) developed a comprehensive scale of 21 items. They identified two dimensions of FLLE—social and private. The social dimension encompasses the enjoyment derived from interpersonal interactions during language learning. The private dimension includes the personal fulfillment and pleasure learners experience when they achieve personal language learning goals. Research by Dewaele and MacIntyre (2016) reinforced FLLE's two-factor structure: social and private. Dewaele et al. (2017) expanded the investigation by including peer-controlled vs. teacher-controlled positivity, a third dimension that highlights classroom dynamics in shaping learners' enjoyment. This study underscores the complexity of FLLE, suggesting that the learning environment and the nature of peer and teacher interactions are crucial in determining the levels of enjoyment experienced by language learners.

Regarding the Chinese context, Li et al. (2018) developed a Chinese version of the FLLE scale to understand the experiences of Chinese high-school students better. They introduced three new dimensions: FLLE-private, FLLE-teacher, and FLLE-atmosphere. The FLLE-private dimension is similar to the private dimension mentioned earlier but might be more specific to the Chinese learning context, including enjoying challenges, successes, and more. The FLLE-teacher dimension emphasizes the impact of the teacher's attitudes on students. The FLLE-atmosphere dimension considers the overall learning atmosphere in the Chinese classroom. Similarly, Jin and Zhang (2021) adapted the scale and examined three dimensions: the enjoyment of teacher support, student support, and foreign language learning. Their path analysis further demonstrated that the enjoyment of teacher and student support indirectly affects language achievement by enhancing the enjoyment of foreign language learning. When students feel supported by their teachers and peers, they are more likely to enjoy the learning process, leading to better language performance (Huang, 2023; Liu and Zhou, 2024).

Empirical evidence highlights FLLE's link with a positive environment, teacher-student support, and peer interactions. Investing in FLLE's multifaceted structure could provide in-depth information about its function, render pedagogical implications for effective learning, and foster learners' proactive engagement in foreign language learning (Dewaele and MacIntyre, 2014, 2016; Jin and Zhang, 2021; Li et al., 2018).

### 2.3 Gamification in foreign language learning

Gamification enhances learners' enjoyment and creates interactive experiences (Cho and Castañeda, 2019), expanding on the significance of a positive learning environment and the proactive engagement of learners in foreign language education. Further, Kapp (2012) describes gamification as applying games' mechanics and strategic thinking to inspire engagement, drive action, enhance learning, and address problems. It uses points, badges, and leaderboards to create game-like experiences (Landers et al., 2015). Studies highlight gamification's benefits for language skills (Loewen et al., 2019; Rachels and Rockinson-Szapkiw, 2017; Redjeki and Muhajir, 2021). Specifically, gamified activities boost vocabulary retention and learner autonomy (Panmei and Waluyo, 2022; Saleh and Althaqafi, 2022), which improves students' performance (Khazaie and Dastjerdi, 2015). Regarding speaking, gamification facilitates proactive verbal engagement and substantial linguistic production, inherently enhancing communicative abilities (Homer et al., 2018; Reitz et al., 2016). Moreover, pronunciation and grammar can be enhanced through a gamifying design with substantial practice and production (Barcomb and Cardoso, 2019; Hong et al., 2022), which improves learners' foreign language accuracy (Castañeda and Cho, 2016). These benefits suggest a pedagogical shift toward interactive learning. These studies (Fahandezh and Mohammadi, 2021; Zou, 2020) confirmed gamification's positive role in improving learners' academic performance.

In addition to its positive impacts on speaking, pronunciation, and grammar, gamification also significantly promotes foreign language reading. A study by Ronimus et al. (2014) found that students' reading comprehension and speed significantly improved when incorporating game elements like virtual rewards and progress tracking into reading courses. By integrating game elements such as points, badges, and leaderboards, students experience more enjoyment and motivation in reading learning, leading to increased active participation (Qiao et al., 2023). During the reading process, gamification elements can stimulate students' competitive awareness, urging them to enhance their reading levels for better leaderboard rankings (Qiao et al., 2024). Competition boosts reading engagement and autonomy (Qiao et al., 2022). In cooperative reading, students share strategies and learn mutually, enhancing their reading abilities (Chen et al., 2020).

Gamification enhances motivation and engagement (Chen, 2021; Dehghanzadeh et al., 2021) and self-regulated ability (Li et al., 2022b), which builds on enhancing foreign language achievement. Further, Li et al. (2021) found that the flow experience could positively impact students' concentration and intrinsic motivation. Reinders and Wattana (2014) further confirmed the role of group-based games in student confidence and WTC, which may positively influence foreign language learning (Sailer et al., 2013; Zhang and Huang, 2023). Additionally, gamification elevates students' enjoyment in learning a foreign language (Cho and Castañeda, 2019; James and Mayer, 2019), subsequently fostering a sense of achievement (Bicen and Kocakoyun, 2018).

While the educational sphere widely acknowledges gamification's diverse benefits in fostering language skills and the psychological dimensions of learning, it is not without its share of controversy and debate within the educational community. Reynolds et al. (2021) argued that gamification positively impacted learners' motivation but with no significant difference in vocabulary learning between gamified and non-gamified groups. Regarding the affective aspects of the learning process, Buckley and Doyle (2014) and Chen et al. (2022) have highlighted such challenges as increased anxiety and limited communication opportunities in competitive settings. Although sustained research has revealed that gamification can engage learners, individual gamified tasks cannot boost students' WTC (Orsatti, 2017).

In conclusion, while prior research on gamification in foreign language learning and FLLE has provided a foundation, notable limitations remain. Most studies have focused on short-term skill boosts and immediate learner responses, neglecting the long-term viability of gamification's impact on language proficiency. Cross-culturally, Western-centric investigations dominate, leaving a dearth of understanding regarding its application and efficacy in non-Western, especially Chinese, educational settings. Additionally, while the importance of FLLE has been recognized, its detailed role in the context of gamified classrooms, especially regarding how it is affected by such environments and influences reading skills, has not been thoroughly investigated. These gaps in the literature lead to the following research questions:

RQ1: To what extent does the gamified classroom affect students' reading proficiency?

RQ2: How does the gamified classroom affect students' FLLE?

RQ3: What is the impact of developing FLLE on reading proficiency in gamified classrooms?

## 3 Methodology

A quasi-experimental design was used to investigate these effects. Enjoyment and happiness can be influenced when learning a foreign language, and playfulness is a crucial characteristic of a classroom in which learners experience FLLE (Dewaele and MacIntyre, 2016). Hence, this study predicts a positive correlation between gamified learning environments and FLLE. As FLLE levels can positively influence learners' academic achievement, this study also predicts a positive correlation between Chinese undergraduate EFL learners' FLLE and their reading performance in gamified classrooms.

### 3.1 Participants

Convenience sampling was used to provide preliminary insights (Neuman, 2014). As it was an opportune time to introduce gamification and gauge its influence on foundational language skills, 220 first-year Chinese undergraduate students (144 males and 76 females) in the initial stage of their university education were voluntarily recruited from a provincial key university in central China. All participants were studying EFL to enhance their language skills and overall literacy, and they were non-English majors across various disciplines. They had proficiency levels demonstrated by Chinese Standards of English (CSE) scores ranging from 4 to 6 according to China's Standards of English Language Ability, an official standard used to evaluate Chinese students' English language competency. A targeted questionnaire was deployed before the study to ensure the absence of prior gamification-strategy exposure. It inquired about ranking systems, point accumulation for correct answers and participation, badge rewards for learning achievements, and group activities integrating these elements. Questions such as “Did your English class have a task-point-ranking system for groups?” and “Were there badge awards for learning goals?” were included. The questionnaire was distributed and collected with strict supervision, and participants were guided to answer accurately. Analysis of the responses confirmed no prior gamification experience. Institutional Review Board approval ensured ethical standards and informed consent was obtained from all participants before the study. Participants' identities were anonymized.

### 3.2 Research context

The study was conducted in a mandatory English reading course for first-year non-English majors. As part of a 4-year degree program, this course included five units from *New Standard College English (Book 1)*, a textbook commonly used in Chinese universities. The course aimed to equip students with a basic understanding of everyday topics, such as campus life and health, as well as reading skills (e.g., understanding logic, searching for detailed information, understanding main ideas and key concepts, understanding and comparing different attitudes and opinions, and inferring and predicting). The course required 12 h of in-class instruction per unit. It was taught by two experienced EFL instructors, both holding doctorates in English Literature and ~12 years of English teaching experience, with a decade specifically dedicated to teaching English reading. Over 16 weeks, participants engaged in 180 min of weekly reading instruction.

Teachers attended a 3-h pre-intervention training, with 1.5 h of training each week, to facilitate the experimental group instructors' comprehensive understanding of gamified instructional strategies. The initial session acquainted the instructor with the key elements of gamification, such as group interactions, scoring systems, badges, and leaderboards. The subsequent session detailed the operational rules and procedures for various gamified activities aimed at enhancing reading skills.

### 3.3 Measures

#### 3.3.1 The “Assessment for Learning” English reading diagnostic assessment

“Assessment for Learning” English reading diagnostic assessments were administered in the pre- and post-test phases to examine changes in reading proficiency. Developed independently by the Foreign Language Teaching and Research Press and crafted by renowned Chinese English test authorities, this assessment is a digital tool for assessing English language proficiency and has been extensively applied in many studies (Fan, 2019, 2021; He, 2019; Jin and Yu, 2019, 2023; Sun, 2019). This English reading diagnostic assessment was based on the CSE and the Common European Framework of Reference for Languages. It is intended to evaluate candidates' reading comprehension skills in English online using the “Assessment for Learning” platform for college students whose CSE proficiency scores range from 4 to 6. The assessment included a range of multiple-choice, matching, and judgment questions in addition to standard single-choice questions. These questions assessed various reading micro-skills, including the student's ability to identify specific information, infer the author's intentions, and compare different points of view.

According to the performance reports provided after the assessment, Fan (2021) reported high reliability for the online reading diagnostic assessment on the “Assessment for Learning” platform for university students. She claimed that most candidates accurately understood the information in the reports, which met the learners' needs, and the reported information allowed learners to improve their English reading skills through various learning methods. As students believed that the diagnostic feedback accurately reflected their strengths and weaknesses in English reading learning, Fan's (2021) study used the reading diagnostic assessment and its performance report as an entry point for bottom-up empirical support for the diagnostic assessment's construct validity.

#### 3.3.2 Chinese version of the FLLE scale

The Chinese version of the FLLE scale (Li et al., 2018) was used to gauge participants' enjoyment of English. As stated in Section 2.2, the scale was modified based on the original scale developed in a European context, consisting of 11 items with a newly confirmed three-factor structure (FLLE-private, FLLE-teacher, and FLLE-atmosphere; Li et al., 2018) to measure Chinese students' FLLE. Table 1 presents the 11 items and their sub-dimensions. Each item is scored on a standard five-point Likert scale, with responses ranging from one (“strongly disagree”) to five (“strongly agree”). According to Li et al. (2018), the three sub-dimensions are FLLE-private, which describes the enjoyment of the challenges, successes, and interesting aspects of EFL; FLLE-teacher, which describes teachers' encouraging and supportive attitudes toward EFL learners; and FLLE-atmosphere, which describes the positive environment of EFL learning. Li et al. (2018) stated that a total score of <33 denotes low or no FLLE, between 33 and 44 denotes moderate FLLE, and >44 denotes high FLLE. The present study's reliability analysis of both the first and second distributions revealed high internal consistency for the total scale (α = 0.859/0.886) and its subscales for the FLLE-private (α = 0.811/0.837), FLLE-teacher (α = 0.921/0.911), and FLLE-atmosphere (α = 0.762/0.703) sub-dimensions. The construct validity of the first and second distributions were both acceptable (χ^2/^*df* = 2.536/2.915, CFI = 0.950/0.944, TLI = 0.933/0.925, SRMR = 0.050/0.056, RMSEA = 0.084/0.094).

**Table 1:** Eleven items of the Chinese FLLE scale and their subdimensions.

**Items**	**Subdimensions**
1) I don't get bored	FLLE-private
2) I enjoy it	FLLE-private
3) I've learned interesting things	FLLE-private
4) In class, I feel proud of my accomplishments	FLLE-private
5) It's a positive environment	FLLE-atmosphere
6) It's fun	FLLE-private
7) The teacher is encouraging	FLLE-teacher
8) The teacher is friendly	FLLE-teacher
9) The teacher is supportive	FLLE-teacher
10) There is a good atmosphere	FLLE-atmosphere
11) We form a tight group	FLLE-atmosphere

#### 3.3.3 Student interviews

A criterion-based sampling strategy was employed in the interview sessions to ensure the credibility of the information gathered (Creswell, 2007). Nine participants (six males and three females) in the experimental group were selected based on their pre-test reading assessment scores to represent a diverse range of reading competencies. Three were low-level (CSE 4), three were moderate (CSE 5), and three were advanced (CSE 6), providing a balanced cross-section of abilities within the group.

As open-ended interviews offer an overall perspective and minimize researcher bias (Gall et al., 2003), three open-ended questions were presented to more comprehensively explore the experimental group participants' attitudes toward the gamified classroom from both the learner-internal and contextual dimensions:

1. “What do you think about the gamified course?”
2. “Why did you enjoy or not enjoy the gamified course?”
3. “What is the teacher's role in the gamified course?”

These questions were designed to gauge the participants' subjective experiences and understand gamification's impact on their learning processes and interaction dynamics within the course. This approach ensured a thorough exploration of the students' perspectives and enhanced the depth of the qualitative data collected.

### 3.4 Gamifying strategy intervention and regular reading instruction

The experimental group underwent a 16-week gamification-based instructional program, while the control group took a traditional reading course of equal duration. Both groups used the same textbook to ensure comparability, engaged in identical out-of-class assignments and had equivalent classroom instruction times. The control group's teacher-led course emphasized exam preparation, vocabulary, and grammar. Instruction followed a structured “pre-reading, while-reading, post-reading” approach to teach language points and reading skills. Gamification was not included in the control group's curriculum. Table 2 delineates the instructional methods, focusing on both conditions.

**Table 2:** Reading instruction for the experimental and control groups.

**Instructional focus**	**Experimental group**	**Control group**
**Instructional procedures**	Stage 1: Initial knowledge engagement	Teacher led-in activities
	Stage 2: Explanation of the game rules (e.g., points, badges)	Teacher-centered instruction on reading
	Stage 3: Game play (collaboration and competition)	Students individual/group activities
	Stage 4: Scaffolding (observing emotional responses)	
	Stage 5: Tallying points and publishing the rankings	
	Stage 6: Summary and feedback	
**Instructional responsibility**	Teacher-led instruction in Stages 1 and 2	Teacher-centered instruction (frequently)
	Peer-collaborative learning with teacher in Stages 3 and 4; Teacher-led instruction in Stages 5 and 6.	Students' group activities or individual participation (occasionally)
		Peer discussion during analyzing textbook articles (occasionally)
**Textbook content**	Unit 1: Diary of a Fresher (campus life) Unit 2: The First Oyster (food) Unit 3: The Pickle Jar (family) Unit 4: Improve Your Study Skills (study skills) Unit 5: Walking Your Way to Health (health) Reading skills	
**Input emphasis**	Rules of each game; Peer collaboration gamified activities and teacher support; Explicit instruction of the targeted reading skill.	Text analysis; Language points (e.g., vocabulary, grammar).

The gamification strategy intervention was designed using an MDA framework, or “mechanics, dynamics, and aesthetics” (Kapp, 2012). Mechanics pertain to the rules and systems that govern gamified components, such as scoring and badges. Dynamics describe the behaviors and interactions that arise from these mechanics, including competition, collaboration, and feedback. Aesthetics involve the emotional responses and experiences evoked by the gamified process, such as excitement and engagement. The intervention incorporated activities, points, badges, and leaderboards, which can effectively boost intrinsic motivation and engagement as supported by the broaden-and-build theory (Hamari and Koivisto, 2015). The unit themes were integrated into diverse gamified group activities, fostering teamwork and resource-sharing to achieve common objectives (Kapp, 2012).

During gamified instruction, the students in the experimental group were organized into teams, and each was assigned a unique badge at the beginning of the course. Points were awarded for answering questions, engaging in gamified activities, and completing the homework. Unit-end rankings were determined by team scores displayed on a board that served as a metric for student engagement in a gamified learning environment. According to the theory, these elements are hypothesized to foster positive emotions that are central to enhancing language proficiency.

**Figure 1:**
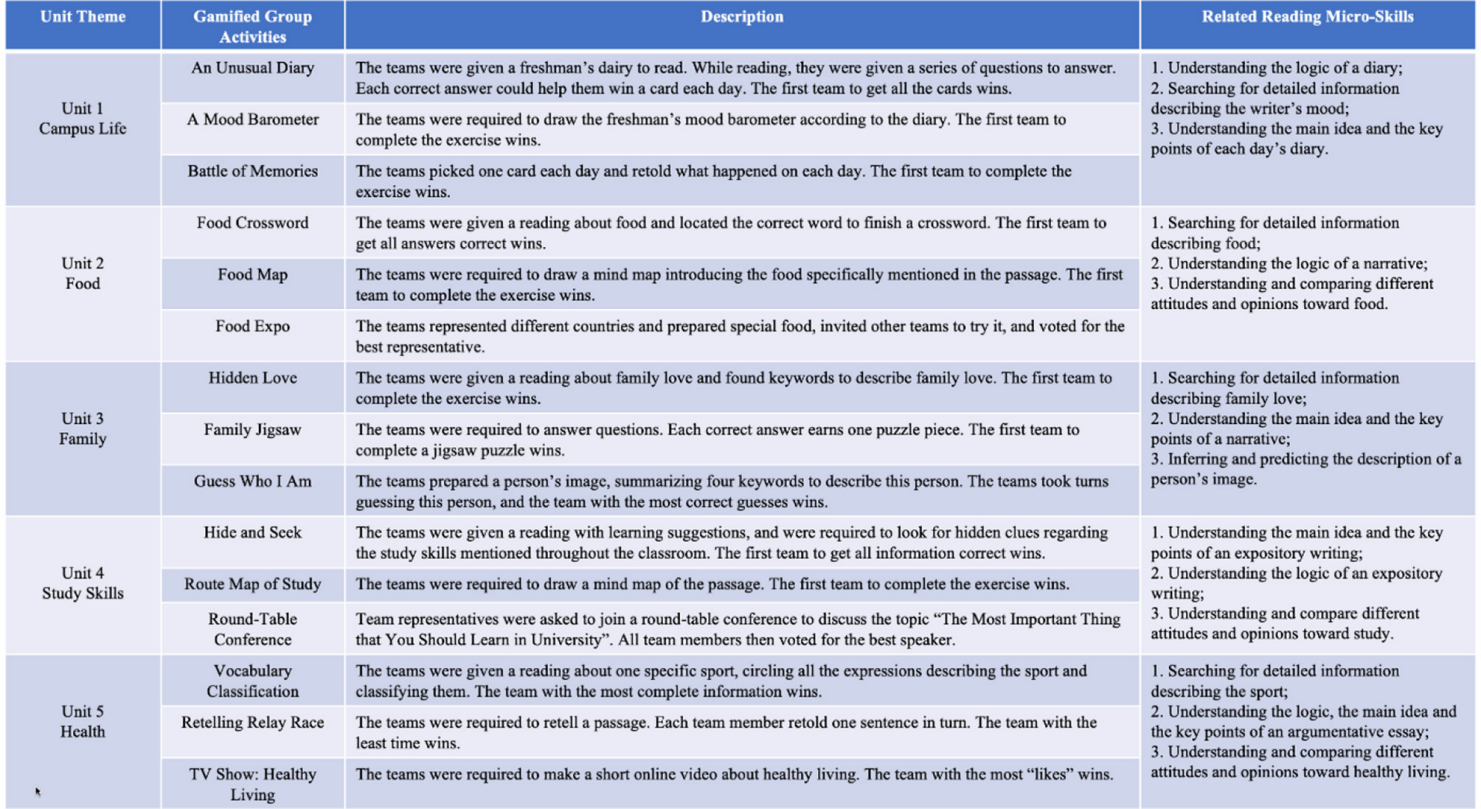
The gamified group activities and related reading micro-skills (each unit).

Figure 1 illustrates that each unit featured three gamified group activities to hone various reading micro-skills. Table 3 outlines the gamification process. For instance, the gamification strategy intervention in the campus life unit began in Stage 1, in which the instructor activated students' prior knowledge related to the targeted reading skill by introducing and practicing the skill. In Stage 2, the instructor detailed the game rules, including the objectives, scoring, and ranking criteria. Stages 3 and 4 involved executing three gamified activities to reinforce topic-related knowledge and reading skills, respectively. In the “Unusual Diary” activity, teams rapidly read text to answer questions, with correct responses earning a chance to win a card representing a day of the week; the first team to collect all the cards won. In the “Mood Barometer,” teams created a mood chart for first-year students based on their diaries, with the fastest team winning. In the “Battle of Memories,” teams recounted daily events, with the quickest team victorious. The top five teams in each activity earned points as a reward. The instructor monitored students' emotional responses to provide assistance and guidance as needed. In Stages 5 and 6, the instructor revealed the game results and facilitated evaluations and reflections on the students' understanding and performance.

**Table 3:** Gamifying strategy instruction procedures (each unit).

Teacher-led instruction	Stage 1: Knowledge activation	Teacher activates knowledge about the activity related to the targeted reading micro-skill.
	Stage 2: Mechanics explanation	Teacher explains the rules of the game (e.g., the goal, points, badges).
Gamified practice with peer collaboration	Stage 3: Dynamics process	Three gamified group activities are implemented to help students practice and memorize the topic-related knowledge and targeted reading skills.
	Stage 4: Aesthetics observation	Teacher observes the students' emotional responses to determine if they need necessary help and guidance.
Teacher-led instruction	Stage 5: Results publication	Teacher publishes the results of the games, and guides them to evaluate and reflect their understanding and performance.
	Stage 6: Summary and feedback	

### 3.5 Data collection

Considering the students' willingness, 106 participants (64 males and 42 females) were placed in the control group, and 114 participants (80 men and 34 women) were placed in the experimental group. The CFLES was administered in Week 1 (pre-test) and Week 16 (pro-test) to investigate students' foreign language enjoyment through the reputable Wenjuanxing platform. Meanwhile, participants were invited to take reading tests on the “Assessment for Learning” platform at the beginning and end of the intervention to examine their changes in reading proficiency. During the intervention, the experimental group participants received a 16-week gamifying intervention (twice a week for 3 h), while participants in the control group received the regular reading course required by the university curriculum and syllabus. During the intervention, the research team minimized any unfavorable consequences for the participants if they missed a class. Nine experimental group students (six males and three females) were invited to follow-up interviews after the intervention to explore students' perceptions of gamification.

### 3.6 Data analysis

After gathering all questionnaires, all effective data were input into SPSS statistical software, version 26, for statistical analyses. Descriptive statistics were calculated, and normality tests were performed. The skewness and kurtosis values in Table 4 indicate that the total scores of all variables in both groups were within the normal range, enabling subsequent parametric tests. Unpaired *t*-tests were conducted to explore RQ1 and RQ2, and one- and three-way ANOVAs were employed to explore RQ3 from a statistical perspective.

**Table 4:** Descriptive statistics and normal distribution of the data.

**Variable**	**Mean**	**SD**	**Skewness**	**Kurtosis**
Change of EFL reading score	0.316	0.670	1.393	2.329
Change of FLLE score in total	3.658	5.949	−0.235	−0.313
Change of FLLE-private score	1.991	3.676	−0.452	0.660
Change of FLLE-teacher score	0.675	1.841	−0.012	−0.874
Change of FLLE-atmosphere score	0.991	2.389	−0.620	0.418

The interviews were meticulously transcribed, producing 14,431 Chinese characters (~7,966 English words). We ensured a comprehensive analysis by adopting a dual analytic approach: a top-down approach guided by the research questions and relevant literature and a bottom-up approach to accommodate emergent themes from the data. This methodological synergy allowed for a balanced consideration of both the theoretical frameworks and spontaneous insights from the participants.

We then systematically input all interview data into NVivo 14, a leading software program for qualitative analyses, to rigorously examine the textual data and conduct a detailed coding process to identify initial codes that were refined into broader themes (Table 5). The coding process strictly followed Braun and Clarke's (2006) guidelines. Specifically, in the initial stage, the first author generated initial codes by carefully reading through the interview transcripts line by line. For instance, when a student mentioned, “I loved the feeling of scoring, and I felt particularly engaged,” the first author coded this as “Engaged, stimulated interest, eager to learn” under the broader theme of “Students' interest in learning in the gamified course.” Similarly, when a student stated, “In the game process, I could review the reading skills without being boring,” it was coded as “Fun (without boring), relaxed, active” within the theme of “The gamified course's learning environment.” This process was repeated for all the data, ensuring no relevant information was overlooked.

**Table 5:** Coding scheme of students' perspectives toward the gamified course.

**Examples**	**Codes**	**Broader themes**
“I loved the feeling of scoring, and I felt particularly engaged.” “This gamified course really stimulated my interest in learning English.” “The gamified group tasks made me eager to learn and review the reading skills.”	Engaged, stimulated interest, eager to learn	The learning interest
“In the game process, I could review the reading skills without being boring.” “I feel relaxed in this class.” “The atmosphere in the classroom is always active, everyone is participating.”	Fun (without boring), relaxed, active	The learning atmosphere
“The teacher was friendly, and her timely help could make me understand the requirements of the game and participate in the game boldly.” “Sometimes some students used non-compliance to finish quickly, and conflicts then arose among students. The teacher needed to mediate in time to maintain the fairness of the game.”	Helpful, guided	The teacher's role
“We completed a variety of games. My predicting and inferring skills have strengthened.” “I would like to speak English more confidently now.”	Reading improvement, confidence	The learning effect

After generating the initial codes, the first author searched for patterns and connections among these codes to identify potential themes. For example, codes like “Engaged,” “Interested,” and “Motivated” were grouped under the theme of “Students' interest in learning in the gamified course” as they all reflected students' positive attitudes and engagement toward the gamified course. The second author then reviewed the initial codes and the proposed themes. They defined and named the themes more precisely based on the content and context of the codes. For instance, the theme that included codes related to the teacher's actions and their impact on the students, such as “The teacher was friendly, and her timely help could make me understand the requirements of the game and participate in the game boldly,” was named “The teacher's role” to convey the essence of this category. Finally, the third author performed a final review to ensure consistency throughout the coding process. To increase the analysis' reliability and credibility, two EFL teaching experts were invited to evaluate the preliminary coding. They provided valuable feedback, and their doubts and questions were addressed promptly. Each theme was further dissected to understand the nuances of students' experiences and perceptions, ensuring that our analysis reflected the complex dynamics of educational interventions.

To more clearly illustrate the occurrence frequency of each theme within the interview data, we conducted a thematic frequency analysis, and the specific results are shown in Table 6. As can be seen from Table 6, learners' internal views of the gamified classroom include the fact that they felt more motivated and engaged. Regarding the atmosphere, most students reported that they enjoyed an active and fun classroom environment. Regarding the teacher's role, students believed that the guidance and help offered by the teacher were essential in the gamified classroom. However, the limited number of participants indicates that the gamified course led to a competitive climate. Learning effects included improved reading skills, learning efficiency, and soft skills, such as team spirit and communication. Several participants mentioned that their confidence and sense of achievement had been enhanced. This frequency data provides additional quantitative support to our qualitative analysis, helping us better understand the prominence and distribution of different themes in the data and further guiding our exploration and interpretation of the research questions.

**Table 6:** Themes in the gamified context.

**Private**		**Atmosphere**		**Teacher**		**Learning effect**	
**Theme**	**Freq**	**Theme**	**Freq**	**Theme**	**Freq**	**Theme**	**Freq**
Motivated	9	Active	9	Guided	9	Reading skill	8
Engaged	8	Fun	8	Amiable	7	Team spirit	8
Enjoyable	8	Relaxed	6	Helpful	6	Communication	5
Concentrated	6	Competitive	3	Essential	4	Confidence	3
Interested	4					Learning efficiency	2
						A sense of achievement	2
Total	35		26		26		28

In constructing a cohesive narrative, we began with the theme of students' interest. The enhanced motivation and engagement increased participation in the gamified learning activities, which affected the learning environment. For example, students' active involvement created a competitive yet collaborative atmosphere. The teacher's role was intertwined with this environment, as they guided and supported the students, ensuring the smooth progress of the games and learning tasks, contributing to the learning effect, as seen in the improvement of reading skills and the development of soft skills.

This deeper approach highlights the meticulous thematic analysis process and underscores our findings' validity and reliability, providing a robust framework for interpreting gamification's effects on language learning.

## 4 Results

### 4.1 Impact of gamified classroom on reading proficiency

**Figure 2:**
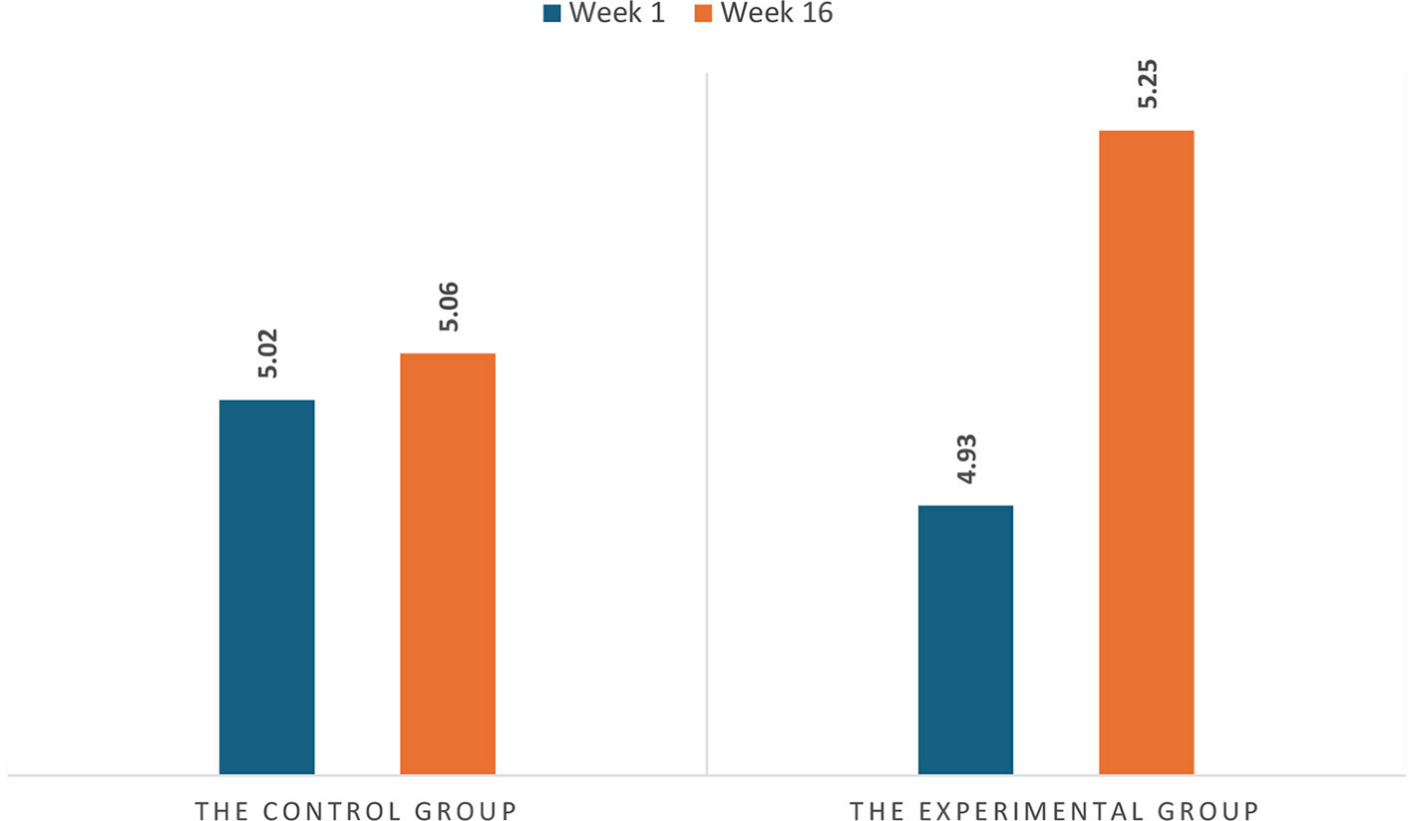
The two groups' reading assessment scores at weeks 1 and 16.

The first research question aimed to explore how gamified classrooms affect students' reading proficiency. The bar charts in Figure 2 present the control and experimental groups' mean reading assessment scores at Weeks 1 and 16. At Week 1, no significant group differences existed (*t* = 1.103, *p* > 0.05), with the control group having an average reading assessment score of 5.02 (in the position of 2 points at CSE 5) and the experimental group at 4.93 (in the position of 93 points at CSE 4), indicating similar initial reading proficiency levels. However, by Week 16, the experimental group exhibited an increase of 0.32 (32 points), while the control group increased by only 0.04 (4 points). As Table 7 demonstrates, the change in assessment scores from Weeks 1 to 16 was significantly greater in the experimental group than in the control group (*t* = 4.023, *p* < 0.001), with a medium effect size, showing that students in the gamified classroom made much more significant progress in reading proficiency.

**Table 7:** *T*-test results of the changes in students' reading assessment scores.

**Variables**	**Group**	***N***	**Mean**	**SD**	***t*** **-test**		
					***t***	**Effect Size (Cohen's** ***d*****)**	**Sig**.
Change of reading score	C	106	0.04	0.31	−4.023	0.530	0.000^**^
	E	114	0.32	0.67			

C, the control group; E, the experimental group.

* *p* < 0.05,

** *p* < 0.01.

Qualitative insights complement quantitative findings. For instance, Participant 6, with significant quantitative improvement (2:47), said, “We completed a variety of games. My predicting and inferring skills have strengthened.” This qualitative feedback directly ties specific gamified activities to enhancing reading skills, thereby enriching our understanding of why the experimental group saw a more significant improvement in reading proficiency, as observed in the quantitative data. Participant 1 (0:36) also stated, “In the game process, I could review the reading skills without being boring,” further exemplifying how the engaging nature of the games and the repetitive practice they afforded made the learning experience effective, thereby potentially enhancing the students' overall reading proficiency. Moreover, the group nature of tasks led to more discussion and sharing of reading strategies. As Participant 9 (3:46) said, “In class, we had to work as a team to complete a variety of reading tasks.” The collaboration contributed to reading proficiency improvement, as evidenced by the quantitative data showing a significant increase in the experimental group's reading scores compared to the control group.

The teacher also played a crucial role in the gamified classroom. As Participant 2 (4:17) noted, “The teacher was friendly, and her timely help could make me understand the requirements of the game and participate in the game boldly. Also, she gave me suggestions to help me improve my vocabulary.” This statement shows how the teacher's support enabled students to engage actively in the learning activities and likely impacted their reading proficiency improvement. Furthermore, Participant 4 (3:52) mentioned, “Sometimes some students used non-compliance to finish quickly, and conflicts arose among students. The teacher needed to mediate in time to maintain the fairness of the game.” This instance highlights the teacher's role in ensuring a positive and fair learning environment, which is vital for the smooth progress of the gamified learning process. The teacher's presence and actions not only provided academic guidance but also contributed to the social and emotional aspects of the classroom, thus facilitating the students' overall engagement and learning experience.

These findings support further exploration of gamification's impact, such as FLLE, which leads us to the following research question.

### 4.2 Influence of gamified classroom on FLLE

**Figure 3:**
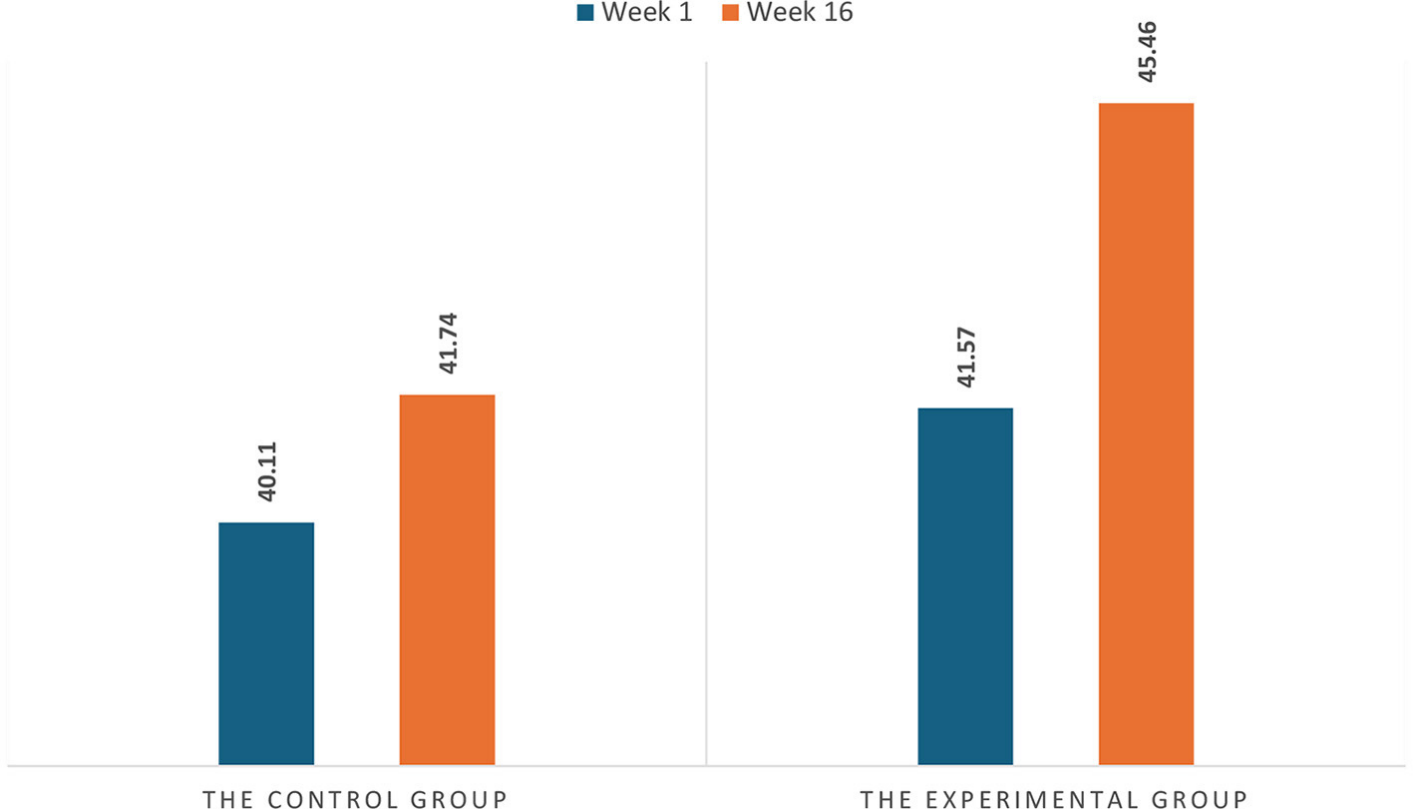
The two groups' FLLE scores at weeks 1 and 16.

The second research question sought to understand how gamified classrooms affect FLLE. Unpaired *t*-tests were used to compare FLLE scores among students. The bar charts in Figure 3 illustrate the control and experimental groups' mean FLLE scores at Weeks 1 and 16. Initially, no significant difference was found between the two groups at Week 1 (*t* = −1.813, *p* > 0.05), with the control group's average FLLE score being 40.11 and the experimental group's being 41.57. By Week 16, the experimental and control groups exhibited increases of 3.89 and 1.62, respectively. As Table 8 indicates, the change in FLLE scores from Weeks 1 to 16 in the experimental group was significantly greater than that of the control group (*t* = −3.491, *p* < 0.01), with a medium effect size. Regarding the three FLLE score sub-dimensions, the change in the experimental group's FLLE-private scores from Weeks 1 to 16 was much greater than that in the control group, while the two groups did not present significant differences in the other two FLLE score sub-dimensions; suggesting that a gamified classroom generally stimulates student enjoyment, especially that generated by learner-internal factors, leading to a more positive FLLE.

**Table 8:** Changes in students' FLLE in 16 weeks.

**Variables**	**Group**	***N***	**Mean**	**SD**	***t*** **-test**		
					***t***	**Effect Size (Cohen's** ***d*****)**	**Sig**.
Change of FLLE score in total	C	106	1.62	3.53	−3.491	0.463	0.001^**^
	E	114	3.89	5.88			
Change of FLLE-private score	C	106	0.59	3.17	−3.008	0.406	0.003^**^
	E	114	1.99	3.68			
Change of FLLE-teacher score	C	106	0.66	2.25	−0.054	0.007	0.957
	E	114	0.68	1.84			
Change of FLLE-atmosphere score	C	106	0.49	1.63	−1.825	0.243	0.070
	E	114	0.99	2.39			

C, the control group; E, the experimental group.

* *p* < 0.05,

** *p* < 0.01.

The qualitative feedback strongly supports these quantitative results, providing a more comprehensive view of the impact of gamified classrooms on FLLE. All interviewees said the gamified course increased their motivation to learn English. Participant 5, with remarkable FLLE improvement (3:27), said, “I loved the feeling of scoring, and I felt particularly motivated in the class when I saw a game won, and the ranking of our group moved up by one place.” Eight participants found the learning process enjoyable, and four became more interested. For example, Participant 9 (0:15) said, “This was the first time I learned English in the class by playing different games, which was interesting.” However, Participant 8 (1:17) mentioned, “I did enjoy the gamified group activities, but sometimes I felt the competition was fierce, and I was a little afraid of our group getting last place. To avoid that, I needed to try very hard, which made me exhausted sometimes.” Despite some concerns from individual students about the competitive aspect, the overall qualitative feedback aligns with the quantitative results, demonstrating that the gamified classroom positively impacted students' FLLE.

FLLE changes in gamified classrooms are significant and seem to be intertwined with the development of reading proficiency. This connection prompts us to investigate the relationship between FLLE development and reading proficiency, as addressed in the third research question.

### 4.3 Relationship between FLLE development and reading proficiency in gamified classroom

The third research question explored the impact of the students' FLLE development on their reading proficiency in a gamified classroom. The results in Table 9 reveal that the development of students' FLLE had a medium effect on the development of reading proficiency (see values of *F, p*, and the partial η^2^). Students' reading scores rose by 0.43 (43 points) with the improvement in their FLLE and fell by 0.01 (1 point) as the FLLE level decreased. Although most participants' FLLE levels improved, seven participants' FLLE levels remained unchanged, while their reading assessment scores increased by 0.39 (39 points). Regarding the three FLLE sub-dimensions, when FLLE-private scores shifted from Weeks 1 to 16 in the gamified classroom, highly significant variances occurred in students' reading proficiency (Table 10). When students' FLLE-private scores decreased, their reading proficiency declined; when their FLLE-private scores increased in the gamified classroom, their reading proficiency improved. This gain and loss indicate that developing students' FLLE levels, particularly the FLLE-private sub-dimension, can positively influence the development of their reading proficiency. Moreover, reading proficiency can improve even if students' FLLE remains stable.

**Table 9:** Influence of the development of students' FLLE on the development of reading proficiency.

	**Development of FLLE**			***F***	***p***	**Partial η^2^**
	**Remain Unchanged (*****n*** = **7)**	**Decreased (*****n*** = **28)**	**Increased (*****n*** = **79)**			
Change of reading score	0.39 ± 0.61	−0.01 ± 0.48	0.43 ± 0.70	4.724	0.011^*^	0.078

* *p* < 0.05,

^**^p < 0.01.

**Table 10:** Three-way ANOVA results for the development of reading proficiency.

**Source**	**Type III sum of squares**	***df***	**Mean square**	***F***	***p***	**Partial η^2^**
Intercept	1.586	1	1.586	3.986	0.048^*^	0.036
Change degree of FLLE-private	6.766	2	3.383	8.500	0.000^**^	0.137
Change degree of FLLE-teacher	0.021	2	0.011	0.027	0.974	0.001
Change degree of FLLE-atmosphere	0.828	2	0.414	1.041	0.357	0.019
Residual	42.583	107	0.398			

* *p* < 0.05,

** *p* < 0.01.

The qualitative findings provide in-depth insights into the relationship between FLLE development and reading proficiency, complementing the quantitative analysis. Students' positive emotional experiences in the gamified course, as described in the FLLE-related qualitative data, led to increased motivation and engagement. For example, Participant 7, with corresponding increases in both FLLE and reading proficiency (3:11), said, “The gamified group tasks made me want to acquire knowledge related to reading skills because by learning those skills, I could contribute to the group score.” Furthermore, the confidence gained in gamified classrooms improved reading proficiency, as several students reported. Participant 9 (3:12) said, “I have made great progress in my English performance in the gamified course, and now I am very satisfied with this,” indicating that the overall positive experience in the gamified classroom, which is related to FLLE, contributed to the improvement in reading proficiency. Additionally, the enhanced confidence and sense of achievement bolstered their motivation and fortified their ability to persevere through challenging reading tasks. For instance, Participant 7 (4:02) shared, “Once I earned one point for my group, I realized that I could also do well in learning English.” Similarly, Participant 3 (2:23) noted, “The gamified group task required us to earn points, but we would not lose any points if we failed, so we could try bravely and not be afraid of failure,” which exemplifies how the non-punitive nature of the gamified environment encouraged students to take risks and engage with difficult materials without the fear of negative consequences. Finally, Participant 2 (2:58) said, “I felt that my hard work in studying was paying off when I got scores. Now I feel confident in learning English,” further illustrating the positive impact of the gamified classroom on students' confidence and their subsequent willingness to confront challenging reading tasks.

## 5 Discussion

This study examined how a gamified classroom improves students' reading proficiency and FLLE. Our study demonstrated that reading proficiency and FLLE both significantly increased. Additionally, the development of students' FLLE positively impacted their reading proficiency within a gamified learning environment.

The first research question focused on how gamified classrooms affect learners' reading proficiency. Both groups' participants improved their reading proficiency by acquiring reading skills. However, participants in the gamified classroom more significantly increased their reading assessment scores (from CSE 4 to 5), echoing a general pattern in the literature on gamification's positive influence on EFL learners' academic performance (Fahandezh and Mohammadi, 2021; Ronimus et al., 2014; Zou, 2020). The gamified design included challenges and rewards tailored to reading tasks, compelling students to engage more frequently and attentively with the reading materials. For example, in the process of the “Food Crossword” activity (Unit 2), students became more adept at noticing specific details in the text, such as the names of different food items, their characteristics, and how they were described, enhancing their ability to understand and extract important information from the reading. Consequently, their overall reading skills were enhanced, which aligns with the fundamental principle that increased exposure and active engagement with reading content can lead to proficiency gains (Allington and McGill-Franzen, 2021; Baek et al., 2020).

Beyond the direct impact, learner engagement also played a significant yet indirect role in improving reading abilities. The gamified classroom incorporated collaborative group activities and immediate feedback mechanisms to boost learner engagement. In the “Retelling Relay Race” activity (Unit 5), students were required to summarize the key points and retell the text in an organized and coherent way. The feedback from their peers and the teacher during the relay race motivated them to perform better. Through qualitative analysis of student interviews, it was found that students actively discussed strategies and clarified doubts. This finding is consistent with Reynolds et al.'s (2021) results that gamification can positively impact students' motivation and further supports Chen et al.'s (2020) study.

Moreover, our findings align with Khazaie and Dastjerdi's (2015) observation that collaborative learning contexts can enhance foreign language performance. The development of soft skills like communication and teamwork accompanied the students' improved reading skills. Take the “Family Jigsaw” activity (Unit 3) as an example; students attentively read the text to discover the answers to the questions and practiced searching for detailed information. The skills of understanding the main idea and key points were also improved as they had to piece together the overall story of the family based on the information they gathered. This collaborative knowledge construction enriches their understanding of the text and promotes the development of higher-order thinking skills, such as analysis and synthesis, which are essential for proficient reading (Lu et al., 2021; Nappu and Hambali, 2022). The collaborative nature of the activity compelled students to communicate and share their findings. Through this process, their communication skills were enhanced as well, confirming Zhang and Huang's (2023) findings on group interaction and communication willingness but contrasts with Orsatti's (2017) study, in which gamification did not effectively promote communication, possibly because of the different gamification formats emphasizing individual rather than group-based activities.

The second research question explored how gamified classrooms affect learners' FLLE. FLLE scores increased from moderate (33 to 44) to high (above 44), indicating enhanced enjoyment and emotional engagement within the gamified classroom. While reflecting and expanding on the findings of James and Mayer (2019) and Cho and Castañeda (2019), our findings also parallel (Bicen and Kocakoyun, 2018). In the qualitative findings, students described how the gamified elements made them feel a sense of achievement and excitement. This emotional boost translated into increased motivation and engagement, as seen in the significant increase in the FLLE-private sub-dimension scores, representing individual emotional engagement and motivation. This finding is similar to the conclusions from Sailer et al. (2013). However, some students reported competitiveness in the interviews, especially for the teams that failed to win. Although the students' FLLE level improved, this fierce competitiveness system might affect their enjoyment, echoing Qiao et al.'s (2024) finding that students may feel inferior if ranking lower on the leaderboard. By comparing with Buckley and Doyle (2014) and Chen et al. (2022), it was found that the level of competition needs to be carefully managed in a gamified classroom.

In our study, the teacher played a crucial role in guiding and helping students, as indicated by the increasing FLLE teacher scores. The teacher interventions maintained a positive atmosphere. This result reflects a general pattern in the literature that teachers' positive traits can influence changes in students' FLLE levels (Dewaele et al., 2019; Dewaele and Li, 2021; Dewaele et al., 2022; Li, 2022). For the teams that did not win in some activities, teachers comforted them. They encouraged them that they still had opportunities to win in the other activities, thus enhancing their learning motivation.

The final question investigated the potential relationship between students' growth in FLLE levels and reading proficiency in a gamified learning environment. The positive impact of growth in students' FLLE levels on their reading proficiency aligns with the conclusions of Li et al. (2019) and Botes et al. (2022). It supports Qiao et al.'s (2023) finding that students experienced more enjoyment while learning reading. However, while the students' FLLE levels remained unchanged, their reading assessment scores improved, indicating that FLLE is not the only factor influencing EFL learners' reading proficiency. As shown in Li et al.'s (2022b) study, gamified learning systems can help students develop an interest in and strategies for self-regulated learning. In our study, group tasks improved time management and self-regulation and enhanced reading proficiency. This finding is consistent with Qiao et al.'s (2022) conclusion.

In addition to reading proficiency, learning outcomes should be considered. The learning effects, including the improvement in communication and team spirit reported in the interviews, reflect the general pattern that FLLE could positively affect students' WTC (Li et al., 2022a; Reinders and Wattana, 2014). In the qualitative findings, students mentioned specific instances where their enhanced FLLE led to more confident communication in the classroom and during group activities, consistent with Oxford's (2016) statement that cultivating positive emotions could be conducive to shaping a supportive learning environment and fostering social connections. Meanwhile, learning efficiency improved in the gamified classroom, which aligns with Jin and Zhang's (2021) finding that learning efficiency can be enhanced when students experience enjoyment in the classroom.

Finally, our study synthesizes these observations into a broader theoretical contribution, illustrating how broaden-and-build theory can be applied to explain the efficacy of gamification in educational settings. Our findings confirm Jin and Zhang's (2019) conclusion regarding the role of enjoyment in building language resources. By enhancing FLLE, gamified learning environments contribute to better language learning outcomes and improved interpersonal skills and personal confidence, enriching educational experience at multiple levels. This finding aligns with the literature showing that positive emotions stimulate students' vitality, eagerness, focus, and communicative competence, which, in turn, facilitate language proficiency acquisition (Fredrickson and Branigan, 2005; Hiver et al., 2021; Leung et al., 2019; Ronnel et al., 2015).

These findings offer new insights into effective pedagogical strategies that promote the integration of gamified elements into language education to enhance both academic and emotional learner outcomes. They highlight the transformative potential of gamification in EFL contexts and underscore the need for further research on the dynamic interactions among gamification, learner engagement, and language proficiency development. However, this study has several limitations. First, its scope was limited to the Chinese context. Second, this study lasted only 16 weeks; therefore, the long-term impact on educational success must still be assessed. Third, the potential influence of gender was not comprehensively examined in this study. Future research could extend to other regions to enhance generalizability, conduct longer-term studies to assess lasting impacts and explore gender differences with more balanced samples and refined designs.

## 6 Conclusions

This study was designed to investigate the impact of a gamified reading course on students' reading proficiency and FLLE. Quantitative results showed greater FLLE improvements in the gamified group than in the control group. This improvement in reading proficiency can be attributed to multiple factors. The collaborative group activities enhanced peer learning and motivation, with students actively discussing reading strategies and clarifying doubts supported by qualitative insights. Immediate feedback enabled prompt adjustments. These elements foster reading skills.

Regarding FLLE, the gamified classroom significantly increased scores, especially in the FLLE-private dimension. Qualitative data indicated that leaderboards and reward systems boosted students' achievement and motivation. Meanwhile, the teacher's management of competition and provision of support were crucial for a positive atmosphere. The positive relationship between FLLE development and reading proficiency was established, though reading proficiency could also improve when FLLE was stable, suggesting other influencing factors.

In conclusion, the findings support gamification's role in academic and emotional learning. Gamification enhances reading skills, enjoyment, and motivation, and teacher guidance prevents excessive strain. Future studies should focus on key elements that drive the success of gamification in language learning, exploring its application in diverse settings to optimize its implementation further and maximize its benefits.

## Data Availability

The original contributions presented in the study are included in the article/supplementary material, further inquiries can be directed to the corresponding author/s.
